# UK food and nutrition security during and after the COVID‐19 pandemic

**DOI:** 10.1111/nbu.12485

**Published:** 2021-02-19

**Authors:** M. Rivington, R. King, D. Duckett, P. Iannetta, T. G. Benton, P.J. Burgess, C. Hawes, L. Wellesley, J. G. Polhill, M. Aitkenhead, L.‐M. Lozada‐Ellison, G. Begg, A. G. Williams, A. Newton, A. Lorenzo‐Arribas, R. Neilson, C. Watts, J. Harris, K. Loades, D. Stewart, D. Wardell‐Johnson, G. Gandossi, E. Udugbezi, J.A. Hannam, C. Keay

**Affiliations:** ^1^ The James Hutton Institute Aberdeen Aberdeen UK; ^2^ Chatham House London UK; ^3^ Cranfield University Bedford UK

**Keywords:** biodiversity, climate change, COVID‐19, food security, nutrition security

## Abstract

The COVID‐19 pandemic is a major shock to society in terms of health and economy that is affecting both UK and global food and nutrition security. It is adding to the ‘perfect storm’ of threats to society from climate change, biodiversity loss and ecosystem degradation, at a time of considerable change, rising nationalism and breakdown in international collaboration. In the UK, the situation is further complicated due to Brexit. The UK *COVID‐19* *F* *ood and* *N* *utrition* *S* *ecurity* project, lasting one year, is funded by the Economic and Social Research Council and is assessing the ongoing impact of COVID‐19 on the four pillars of food and nutrition security: access, availability, utilisation and stability. It examines the food system, how it is responding, and potential knock on effects on the UK’s food and nutrition security, both in terms of the cascading risks from the pandemic and other threats. The study provides an opportunity to place the initial lessons being learnt from the on‐going responses to the pandemic in respect of food and nutrition security in the context of other long‐term challenges such as climate change and biodiversity loss.

## A worsening ‘perfect storm’

In 2009, Sir John Beddington, then Chief Scientific Adviser to the UK Government, made his famous ‘A Perfect Storm by 2030’ statement (Beddington 2009) on risks of civil disruption arising from coinciding food shortages (demand increases by 50%), insufficient energy (demand up 50%) and water scarcity (demand up 30%) at a time of climate change and increasing world population (anticipated to reach 8 billion by 2023). Since then, increased understanding of the risks associated with climate change (IPCC 2018, 2019), the degradation of the natural environment (IPBES 2018, 2019) and species extinction (Ceballos *et al*. 2020) has added to our appreciation of the additional threats posed to food and nutrition security by ecosystem service deterioration. The ‘storm’ analogy implies a short period of disturbance and a calm afterwards, whereas what we face are long‐term major societal perturbations and risks from multiple causes and therefore the need for new strategies, pathways and adaptations. A key element in understanding the risks posed to food security by climate hazards is the relationship between extreme weather and its impacts on disruptive shocks that cascade across the sectors and borders to drive up prices and change availability of food in any country (CCRA2 2017; Challinor *et al*. 2018). An example of such cascading risks – likely with an underlying climate signal – is the emergence and spread of new human diseases. There is increasing recognition that the causes of the pandemic, the estimated economic costs of which globally range between $8‐16 trillion, are closely related to human encroachment on the natural environment, biodiversity loss, climate change and the structure of our food system (IPBES 2020).

The COVID‐19 pandemic is now causing impacts on global health, economies, consumer behaviour and the processes by which food is produced and distributed. It is highlighting both the strengths and weaknesses of the global food system. Whilst these coinciding pressures and risks imply a ‘perfect storm’ scenario in 2020, it can also be viewed as a unique opportunity to progress a range of actions to support the UN Sustainable Development Goals and redevelop our relationship with the environment and how we work with nature to meet our physical and wellbeing needs.

The overall unprecedented situation for the UK in terms of food and nutrition security is, however, even more complex given coinciding developments that will have strong and lasting influences on trade and food production and hence food and nutrition security. These include the following:

- Brexit, the EU‐UK Trade and Cooperation Agreement and other trade negotiations and role of the World Trade Organisation (WTO) in determining future trade regimes and standards in food.
- Replacement of the Common Agricultural Policy with The Agriculture Bill (2020) (https://services.parliament.uk/Bills/2019‐21/agriculture.html) and Environment Bill (https://services.parliament.uk/Bills/2019‐21/environment.html) in Parliament and devolved government equivalents influencing what, where and how food is produced, how land is managed and what support producers receive.
- Net zero carbon emission targets that influence what land is used for and how it is managed [*e.g*. 30 000 ha tree planting target by 2025, and an increase to 30% (400 000 ha) of the English countryside with protected area status by 2030 (*BBC News* 2020), as well as other demands on land (house building, roads etc.)].
- An increased awareness of vicious circles that can link poverty, poor diets and poor health.
- Changing environments that may impact farming (soil degradation, declining insect populations and reduced ecosystem services).
- Climate change impacts on UK production (*e.g*. yield fluctuations through changing weather, particularly extremes of rainfall and temperature and increased incidence of pests and diseases) (CCRA2 2017). Climate change, and particularly the hazards arising from changing weather patterns, also has the potential to rapidly affect market dynamics (supply chain disruption, prices and availability of both foods and agricultural impacts) impacting UK agricultural practices.
- These developments are occurring at a time of economic slowdown and interacting with changing societal beliefs, attitudes and behaviours towards food (both here and abroad, where significant drivers on UK food systems have leverage), the environment and international relations.

## Food systems and food and nutrition security

The global food system provides critical underpinning support for society and represents the clearest connection between human needs and the ability of the environment to provide them. The food system has undergone considerable change in recent decades, with globalisation and international food trade facilitating the consumption of new products, access to the same foods all year round rather than seasonal foods (Lang 1999; *The Guardian* 2014), and cheap energy‐dense, nutrient‐poor foods. Consumption (particularly meat and dairy) has increased but so has the concentration of global energy intakes, through a few staple crops (*e.g*. wheat, rice, soya and maize) (Antonelli *et al*. 2020). Prior to the pandemic, this broad configuration of the food system was viewed as having both strengths and weaknesses. Trade helps build stability in international relationships and creates employment and opportunity to improve standards of food safety, but it is also a source of risks to local food security if disruption occurs [*e.g*. due to climate change (GFSP 2015)]. However, there are large inequalities: ‘*Nearly every country in the world faces serious health problems linked to the consumption of either too little nutrient‐rich food or too much energy‐dense food*’ (IFPRI 2015). Inequalities, lack of access to affordable food, and rising obesity and malnutrition have been concerns for some time in the food system arena in the UK as in many other countries and international organisations. Hence, prior to the pandemic, there were concerns about the UK food system and calls for transformation of consumption patterns to improve diets and health and to achieve greater resilience and sustainability in production and supply chains (Williams *et al*. 2018; Lang 2020; Boelsen‐Robinson *et al*. 2020; Springmann *et al*. 2020). There have been calls for radically transforming the global food system to achieve a resilient food system (Willett & Rockström 2019; Poore & Nemecek 2018; Hawkes 2020), and even more so now during the pandemic (Stordalen & Rockström 2020). The pandemic recovery presents opportunities to better align policy responses that improve UK food system resilience, result in healthier diets and contribute to the achievement of global Sustainable Development Goals.

The pandemic is also forcing a re‐evaluation of what we mean by food system resilience – ‘resilience for whose benefit: the food industry or consumers (and within each of these groups)?’ and ‘what does resilience look like, and how do we achieve it?’ The food system has evolved, certainly in developed nations, to one focused on economic efficiency (and does not consider externalities such as ecological or health costs) rather than on resilience and sustainability. The ‘just in time’ value chain approach has greater exposure to threats, such as the pandemic, and can be seen as brittle in terms of resilience as it may be relatively easily disrupted or have supply chains broken (Parsons 2013; Benton 2019). The pandemic has increased online shopping and consumer choices are altering (Waitrose 2020; *The Guardian* 2020), illustrating how quickly behaviours can change.

COVID‐19, as a systemic shock that has critically impacted food systems, highlights the need to consider resilience as a guiding principle. This is recognised by Henry Dimbleby in the National Food Strategy’s first report (Dimbleby 2020):

‘There is a lot of work to do if we are to rebuild a food system that delivers safe, healthy, affordable food to everyone; that is a thriving contributor to our urban and rural economies; that restores and enhances the natural environment for the next generation; that is built upon a resilient, sustainable and humane agriculture sector; and **that is robust in the face of future crises**’ [Emphasis ours] (Dimbleby 2020, National Food Strategy Part One, P.17).

The pandemic recovery will be challenging, but given the additional and mounting climate and biodiversity challenges, there is also an opportunity to rebuild the food system and address wider threats to society.

## About the project

This ‘rapid response’ project consists of a consortium led by The James Hutton Institute in collaboration with Chatham House (Royal Institute of International Affairs) and Cranfield University.

The objectives are to:

- Assess the immediate response of current and post‐pandemic global food systems.
- Assess UK food system responses and vulnerabilities.
- Assess cascading causation of further impacts within a common framework of differing plausible scenarios.
- Develop scenarios for alternative UK agricultural land use, land management and supply/value chain relationships to better understand the consequences for food system resilience and long‐term environmental sustainability, both in the UK and overseas.
- Identify spatial environmental consequences of pandemic responses and opportunities for improved food and nutrition security and food system resilience through sustainable agriculture.
- Review lessons learned from the pandemic for adapting the food system to help achieve climate change and biodiversity goals.
- Provide evidence‐based recommendations to inform policy development to increase food system resilience and sustainability.

As health and economic impacts are altering supply, distribution and demand, this research was designed in recognition that post‐pandemic crisis recovery programmes need to ensure food and nutrition security whilst also supporting climate change mitigation and adaptation, biodiversity and ecosystem resiliency objectives.

This project will provide government, business and other decision makers with evidence to help develop robust food systems that deliver food and nutrition security and which are better placed to respond to the current pandemic and future risks and opportunities.

There are seven key deliverables:

Deliverable 1. A risk assessment of the global food system considering how direct and indirect COVID‐19 impacts and responses are propagating risks to food and nutrition security via multiple system components. Data used are from various sources of official statistics, media reports, key informants, academic and grey literature, and Chatham House’s own resourcetrade.earth database (Chatham House 2020) of global commodity trade flows and prior quantitative assessment of Chokepoints and Vulnerabilities in Global Food Trade. A robust conceptual framework is used to assess risk cascade mechanisms (building on the CASCADES 2020 framework) and transmission pathways in the food system with a detailed understanding of contemporary market dynamics affected by the pandemic. Critical food system pathways are identified, including supply‐chain and infrastructural chokepoints threatening the UK’s food and nutrition security. The UK’s role in improving the resilience and sustainability of both its own food and nutrition security in the face of COVID‐19 and, through co‐ordinated action and leadership, the global food system is considered. Data gaps and capacity constraints to monitor real‐time emerging situations are also identified to improve future research and post‐pandemic analyses.

Deliverable 2. A risk assessment of UK food system responses and vulnerabilities and consequences on access, availability, utilisation and stability. This contextualises global drivers and pressures from a UK perspective. This will characterise and assess supply and demand side responses from agricultural value chains including producers, distributors, retailers and consumers, using an online survey and literature review. The impacts on UK exports and responses in the UK food production, such as intensification increases and impacts on the environment will be assessed.

**Figure 1:**
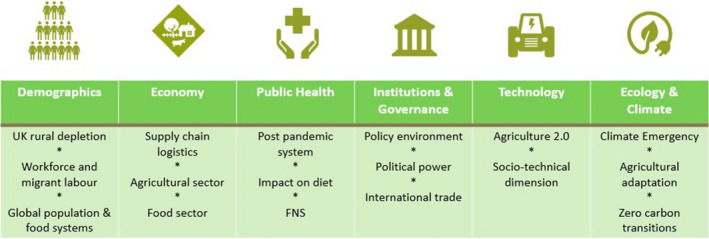
Factors considered within plausible scenario development: Six Drivers of Change that are critical uncertainties. FNS, food and nutrition security.

Deliverable 3. Develop a framework for plausible scenario building (Benton 2019) and assessment of cascading risks and causation, to explore ‘what if…’ questions to identify further future risks, potential mitigation strategies, and opportunities to improve food and nutrition security and food system resilience. This deliverable uses a novel, hybrid DELPHI/participatory scenario development method to apply social science risk frameworks (Beck 2006) to analyse scenarios. The framework uses a morphological approach (considering more than two principle drivers of change with more than two possible assumptions, hence not simply a 2 × 2 matrix, instead we use 9 drivers and 5 assumptions) and builds on the concept of using multiple drivers (economy, demographics etc., see Fig. 1), impact shocks and cascading impact transmission scales, pathways and responses. This is a concept adapted from the approach used to assess climate risks (CASCADES 2020). The concept of plausibility is often preferred over that of probability by scenario planners specifically when the ambition is to strategically explore future possibilities, looking to seize opportunities and avoid risks, rather than make forecasts. It also aligns with more qualitative than quantitative or probabilistic scenario planning. While both approaches have their merits (Ramirez & Selin 2014), choosing plausible, normative outcomes allows greater flexibility to move beyond current assumptions. This is useful when attempting to make flexible plans rather than attempting to determine what the future will most likely be like.

Deliverable 4. Assess opportunities and constraints of UK food and land management options in terms of production potential and environmental impacts. This explores options for changes to food production and land management in the UK, in the context of the plausible scenarios and a new focus on resilience‐building. This task will identify over‐ and under‐represented food groups required for healthy diets, and assess the opportunities and risks if UK domestic food production were more closely aligned to UK demand for food (*e.g*. increased production of higher fibre starchy foods, legumes, vegetables, fruits and reduced production of red meat). The questions to be examined include the effect on UK livestock production if there was no imported feed, and the impacts of agro‐ecological farm practices (*e.g*. organic, conservation or regenerative agriculture) which can have local positive environmental benefits (Burgess *et al*. 2019) but potentially global negative effects if production is increased elsewhere (Smith *et al*. 2019). The capabilities of and capacity for new technologies (*e.g*. protected cropping and vertical farming) and opportunities for rapid up‐scaling will also be assessed.

Deliverable 5. Spatial assessment for the UK of the impact of food and land management change options. This effort recognises that the food types and associated land management options are constrained by the UK’s soils and weather (including inter‐annual variations), socio‐economic consequences and environmental impacts. The aim is to identify spatial consequences on the environment of pandemic responses and opportunities for improved food and nutrition resilience through sustainable agriculture, providing a spatial assessment of the impact of food and land management change options. The research builds on relationships of land use responses to healthy diets, income, domestic sourcing and technology (Williams *et al*. 2018) and uses existing land use and capability mapping (Keay *et al*. 2014) to spatially explore and quantify the anticipated consequences of alternative land use options, considering greenhouse gas (GHG) commitments and environmental and biodiversity targets. Baseline UK maps of current land use and commodity production will be used to provide a ‘Business as Usual’ counterfactual. The spatial impact of land use options, within contrasting plausible scenarios, on food production and key economic (*e.g*. import and exports) and environmental indicators (*e.g*. carbon sequestration; soil degradation) will be determined using modelling tools (Morris *et al*. 2005; Graves *et al*. 2015; Smith *et al*.^2018^, ^2019^), with appropriate assumptions made regarding climate change, inter‐annual weather variations, and the incidence of crop and livestock diseases.

Deliverable 6. Reviewing lessons learned from the pandemic to improve UK Food and Nutrition Security. This is to collaboratively assess and interpret what we can learn from government, industry and society’s responses to the pandemic to identify strengths and weakness in the global and UK food system. Through dialogue with stakeholders and online workshops and building on project findings, the lessons learned and how perceptions of risk have been affected will be reviewed. Lessons will be interpreted in the context of plausible future pandemics and other tangible risks to the food system including climate change, biodiversity and habitat loss and soil degradation.

Deliverable 7. Dissemination events (workshops and reports) on how the food system has coped with the pandemic and recommendations regarding what changes will help enable the food system to become more resilient and achieve food and nutrition security.

## Global COVID‐19 impacts

Thus far (January 2021), UK imports of food, drink, animal feed and agrochemical inputs have largely remained stable throughout the pandemic, though airfreighted fruit and vegetable imports have experienced greater disruptions, particularly during the height of the initial lockdown (King & Wellesley 2020). Equally, agricultural input prices have remained largely stable throughout the pandemic suggesting few supply constraints, whilst farmgate prices have risen for arable goods and contracted for meat, suggesting suppressed demand. Experimental consumer price data suggests price inflation during the height of the March‐April lockdowns and deflation thereafter. Globally, to date, the pandemic has had differentiated geographical impacts. Whilst there are some concerning supply‐chain constraints in some regions and some significant price rises in some markets, generally food supply is plentiful and impacts have mostly been the result of demand contractions. Some countries have implemented food and agriculture trade measures, generally to restrict exports and liberalise imports, but these have been nothing like as severe or damaging as the unilateral measures adopted during the 2007‐2008 and 2010‐2012 food price crises. Nonetheless, economic pressures resulting from COVID‐19 could yet cause major food crises around the world if people are unable to afford nutritious food. Whilst impacts to date, in aggregate, have been relatively mild, there is little evidence that this is the result of particularly effective or coordinated interventions. The global impacts of the pandemic, particularly the economic effects, will likely affect the UK’s food and nutrition security for the coming years. With a second wave of the pandemic having taken hold, and with some affected planting seasons started it is likely that the full scale of impacts are yet to be fully realised.

## UK impacts

Up to January 2021, there has been a large variation in how the pandemic has impacted the UK’s food system and differentially affected society. The pandemic led to a constant state of change and hence a highly dynamic impact and response set of conditions. At the time of writing this article, the UK was in a third lockdown period. Whilst global food production and trade has enabled imports to remain relatively stable and internal production has maintained sufficient supply to avoid severe issues of food availability, physical and economic access has been severely impacted. This has made more visible the previously identified flaws in the food system of inequalities and variation in diet quality (Lang 2020) in the UK population. Hence, whilst food availability (production and supply) has remained relatively stable, reduced economic access (affordability of food) and physical access has greatly increased the number of food insecure people. The newly redundant or self‐employed people who lost their source of income due to the pandemic exacerbated an already increasing reliance on foodbanks and charity support for low income sections of society. Paradoxically this increase also included people working in the food sector, for example fisherman (***The Guardian*** 2020).

## Future risk and opportunity scenarios

The need to transform food systems to deliver healthier diets, more sustainably, has been a predominant feature of discourse over the last few years (IPCC 2019; IPBES 2019; Swinburn *et al*. 2019; Willett & Rockström 2019). The growing prominence of the systemic nature of the challenges faced has increased awareness of the issues among broader constituencies than the traditional narrow epistemic communities. COVID‐19 has shone an additional spotlight on the need for system transformations that ensure resilience in the face of acute and unforeseen shocks, as well as those that address chronic shortcomings. Resilience has a number of properties, many of which have been eroded within food systems to increase efficiency. These include the following: functional redundancy (*e.g*. food storage in supply chains, rather than ‘just‐in‐timeness’), modularity and decentralisation (rather than centralisation to maximise economies of scale), diversity (whether diversity of sourcing, supply chains, products) and flexibility/adaptability/substitutability (including being able to switch supply chains, production to new products, or accepting changing availability of foods in the supply chain, retail or diets). However, resilience comes at a cost because it is less efficient, whilst it also seeks to account for externalities. Hence, to prevent increasing inequality, resilient food systems need to be supplemented by additional social safety nets, as well as behavioural changes (*e.g*. reducing food waste, avoiding unnecessary purchases).

## Role of agroecology

There are a range of ‘agro‐ecological’ or ‘regenerative’ practices that allow continued agricultural production whilst at the same time increasing the sequestration of carbon and the enhancement of biodiversity at a farm‐scale (Burgess *et al*. 2019). Such practices extend from conservation agriculture and multi‐paddock grazing systems, to agroforestry and the greater use of tree crops, to organic systems and even rewilding. Whilst the environmental benefits of such systems can be demonstrated at a farm‐scale (Burgess *et al*. 2019), reduced yields per hectare can lead to negative off‐farm effects if those reduced yields lead to greater production elsewhere (Smith *et al*.^2019^). However, such effects could be reduced, if changes in agricultural production occur alongside changes in diet and the reduction of food waste.

## UK’s protein import dependency

As an example of the UK’s food system dependencies, here we consider proteins and where and how it is sourced. Food (not including animal feed) imports as a proportion of UK consumption is approximately 45%. However, the UK has imported approximately 70% of its high‐protein grain feed requirement for many years. Whilst this dependency seems high, it is around only 10% higher than total food imports to the UK, which at 64 % [expressed as the ‘self‐sufficiency’ (production/supply ratio)], and this reflects a consistent year‐on‐year decline from 78% (a 14% drop) over the past 35 years (National Statistics 2020). This level of protein import dependency in the UK is similar to that of EU country averages. The demand for feed protein is realised mainly in the form of soybean, which is often sourced from land that had previously been biodiverse, carbon negative rainforest and/or the Cerrado regions of South America (Barona *et al*. 2010). The crop replacements for native ecosystems have greenhouse gas emissions associated with them including carbon dioxide (CO_2_) and nitrous oxide (N_2_O) (Reijnders & Huijbregts 2008). These crop imports serve animal feed industries, especially bovine [which also contribute to methane (CH_4_) emissions] and poultry markets (EC 2019). Even before the impact of COVID‐19, UK protein import dependency has been persistent, despite the consequences on these important South American regions. Ecologically, they have key roles in helping safely regulate biogeochemical cycles (Sampaio *et al*. 2007; Rajão *et al*.^2020^), carbon sequestration and climate change impacts. This continued production of animal feed from locations providing vital ecosystem services has occurred despite the existence of UK and EU home‐grown alternatives (de Visser *et al*. 2014). The cultivation of grain legumes in the UK is very much dominated by combinable (*i.e*. dry harvested) peas (*Pisum sativum* L.) and faba beans (*Vicia faba* L.), although alternative species are now being grown with increasing frequency, *albeit* still at low very levels nationally. These include the following: soybean [*Glycine max* (L.) Merr.]; lupin (*Lupinus angustifolius* L., *L. albus* L.); lentil (*Lens culinaris* Medik.); *Phaseolus vulgaris* L., including common‐ (*e.g*. navy) and French bean types, plus other more novel forms such as runner bean (*P. coccineus* L.)*;* and chickpea (*Cicer arietinum* L.). The very low levels of grain legume inclusion in EU cropped systems [1‐4% of the rotation, including those of the UK (Watson *et al*.^2017^)] falls below levels desired for arable systems [of up to 25% (Iannetta *et al*. 2016)]. Such exclusion also means that the potential environmental benefits of grain legumes to local biodiversity, soil quality and fertiliser replacement (Nemecek *et al*.^2008^) are also forfeit. To reiterate, even before any potential food security risks posed by COVID‐19, there were strong ethical and environmental drivers to source protein from home‐grown legumes. These drivers have been acknowledged in EC‐CAP and going forward the EC’s ‘plant protein plan’ (EC 2018). Increasing the levels of production for home‐grown high protein legume grains has proved recalcitrant historically (Squire *et al*.^2019^), and is compounded by a lack of post‐harvest processing capacities (*e.g*. dehulling and protein:starch fractionation and/or enrichment) and processing technology (such as for protein extrusion). However, recent shifts in consumer diets motivated by ethical, environmental and personal health concerns are already driving major shifts in protein markets (Ebert 2014; Hamann *et al*. 2019), though whether the socio‐economic and environmental potential of legumes are realised ‘at home’ remains to be seen. As such, the UK’s volatile global market‐dependent protein supply has been disrupted by COVID‐19, since supplies of animal feed and increased prices are exacerbated by closure and reduction in services of private (and public) food outlets at home (Choudhury 2020). The extra risk posed by COVID‐19 could be perceived as an opportunity to re‐establish more‐sustainable legume‐based cropped, food and feed systems at home.

## Changing diets and consumer behaviour

The future UK food system will need to adapt in response to dietary change. This may take a number of directions and have different rates of change, dependent on governmental incentives and other drivers. A move to healthier diets in the UK would generally result in the consumption of less sugar and salt, less red and processed meat and more fish, higher fibre starchy foods, fruits and vegetables. Compared to current diets this would tend to decrease on‐farm greenhouse gas emissions, especially from ruminants and potentially release grassland for other uses and marginally increase the demand for cropland, both in the UK and overseas (Williams *et al*.^2018^).

## Food system power relations

An exploration of the impacts of the pandemic on food‐aligned NGOs (including charities) operating in the UK is exploring challenges to how these organisations operate and the increased demand for their services. It is also investigating evidence that in the absence of sufficiently coordinated and effective government responses to food insecurity (particularly for vulnerable people and children), NGOs are increasing their level of coordination, activity and, in some cases, control of food supply chains as an alternative/parallel to the more traditional role of government in looking after the population.

The next few years are likely to see increased calls for radical changes to the food system as coinciding pressures increase. This will add to the already substantial challenges society faces to deal with the climate and biodiversity emergencies. The *COVID‐19 Food and Nutrition Security* project will aim to provide additional insights to inform strategic planning and solution development.
